# Task-Dependent Effective Connectivity of the Reward Network During Food Cue-Reactivity: A Dynamic Causal Modeling Investigation

**DOI:** 10.3389/fnbeh.2022.899605

**Published:** 2022-06-24

**Authors:** Peyman Ghobadi-Azbari, Rasoul Mahdavifar Khayati, Arshiya Sangchooli, Hamed Ekhtiari

**Affiliations:** ^1^Department of Biomedical Engineering, Shahed University, Tehran, Iran; ^2^Iranian National Center for Addiction Studies, Tehran University of Medical Sciences, Tehran, Iran; ^3^Department of Psychiatry, University of Minnesota, Minnesota, MN, United States

**Keywords:** fMRI, cue-reactivity, dynamic causal modeling, obesity, craving

## Abstract

Neural reactivity to food cues may play a central role in overeating and excess weight gain. Functional magnetic resonance imaging (fMRI) studies have implicated regions of the reward network in dysfunctional food cue-reactivity, but neural interactions underlying observed patterns of signal change remain poorly understood. Fifty overweight and obese participants with self-reported cue-induced food craving viewed food and neutral cues during fMRI scanning. Regions of the reward network with significantly greater food versus neutral cue-reactivity were used to specify plausible models of task-related neural interactions underlying the observed blood oxygenation level-dependent (BOLD) signal, and a bi-hemispheric winning model was identified in a dynamic causal modeling (DCM) framework. Neuro-behavioral correlations are investigated with group factor analysis (GFA) and Pearson’s correlation tests. The ventral tegmental area (VTA), amygdalae, and orbitofrontal cortices (OFC) showed significant food cue-reactivity. DCM suggests these activations are produced by largely reciprocal dynamic signaling between these regions, with food cues causing regional disinhibition and an apparent shifting of activity to the right amygdala. Intrinsic self-inhibition in the VTA and right amygdala is negatively correlated with measures of food craving and hunger and right-amygdalar disinhibition by food cues is associated with the intensity of cue-induced food craving, but no robust cross-unit latent factors were identified between the neural group and behavioral or demographic variable groups. Our results suggest a rich array of dynamic signals drive reward network cue-reactivity, with the amygdalae mediating much of the dynamic signaling between the VTA and OFCs. Neuro-behavioral correlations suggest particularly crucial roles for the VTA, right amygdala, and the right OFC-amygdala connection but the more robust GFA identified no cross-unit factors, so these correlations should be interpreted with caution. This investigation provides novel insights into dynamic circuit mechanisms with etiologic relevance to obesity, suggesting pathways in biomarker development and intervention.

## Introduction

With the population of overweight or obese adults reaching 1.9 billion in 2016 (World Health Organization, 2021) and given the immense and growing disease burden associated with high BMI (Dai et al., 2020), sustained effort has been dedicated to elucidating the physiology of overweight and obesity and the neurocognitive architecture underlying relevant behaviors. Considering the central importance of excess calorie intake and food choice patterns in the development of obesity (Kadouh and Acosta, 2017), much of the cognitive neuroscience research has focused on how food is perceived and evaluated; how food craving and hunger are induced, experienced and regulated; and how these and other processes interact to shape eating behavior (Leng et al., 2017; Letra et al., 2017; Stice and Burger, 2019). A critical feature of typical eating behavior is its being closely preceded, mediated, and followed by rich set of associated cues (Jastreboff et al., 2013; Joyner et al., 2017), which may form conditional associations with ingested food (Boswell and Kober, 2016). Once such appetitive conditioning develops, food cues elicit a range of conditioned physiological processes; these include increased parasympathetic tone (Nederkoorn et al., 2004) and salivation (Jansen et al., 2003); neural activity, particularly in reward processing and energy regulation circuits (Tang et al., 2012; Cosme and Lopez, 2020); and cognitive processes such as attention allocation (Liu et al., 2019). Food cues can also induce food cravings (Werthmann et al., 2013) and hunger, even in otherwise satiated individuals (Cornell et al., 1989; Johnson, 2013).

This complex set of conditioned responses to food cues is often termed “food cue reactivity” (Boswell and Kober, 2016). Food cue reactivity and appetitive conditioning are, by themselves, normal and evolutionarily significant enablers of adaptive motivated behavior: in cue-rich environments, they can appropriately motivate food seeking while shifting individuals toward a food-oriented neuro-physiological state in preparation for food choice, ingestion, and digestion (Martin-Soelch et al., 2007; Power and Schulkin, 2008; Leng et al., 2017). However, evidence is mounting that certain patterns of food cue reactivity and their neural substrates contribute to overeating in overweight and obese individuals (Jansen et al., 2003; Havermans, 2013; Ng and Davis, 2013). This maladaptive food cue reactivity is hypothesized to involve a core dysfunction of the hedonic aspect of conditioned food cue reactivity; namely, the misattribution of overwhelming appetitive salience to food cues which would drive cue-induced “wanting” (or craving) of food and subsequent overeating, regardless of how rewarding the food itself is (Val-Laillet et al., 2015; Robinson et al., 2016; Stice and Burger, 2019). Such “incentive sensitization” theories of obesity have parallels in theories of cue-induced craving in drug addiction (Robinson and Berridge, 1993) and are supported by evidence that wanting, hunger and food consumption can be heightened by food cues, independently of “liking” intensity (Joyner et al., 2017; Polk et al., 2017).

In recent decades, rapid advances in the cognitive neuroscience of eating and obesity have been enabled by functional neuroimaging modalities, in studies which typically involve the recording of changes in brain hemodynamics or electrophysiology during exposure to food-related stimuli to probe the neural bases of hedonic cue-reactivity (Leng et al., 2017). Notably, reported observations can generally be interpreted under an incentive sensitization framework: Food cues generally induce heightened prefrontal and amygdalar activation in obese individuals, potentially reflecting the higher valuation or secondary cognitive responses to such valuation, and alterations in the ventral tegmental-striatal dopaminergic signaling suggest that core dopaminergic processes may underly the aberrant incentive sensitization (Carnell et al., 2012; Val-Laillet et al., 2015; Moore et al., 2017; Devoto et al., 2018). Neural food cue reactivity research may have clinical utility far beyond etiological explanation: the identification of mechanistic neural underpinnings of dysfunctional eating and food choice in obesity can suggest interventions to reduce cue exposure (Boulos et al., 2012), regulate craving (Kober et al., 2010) or extinguish conditioned cue responses (Frankort et al., 2014); and based on a recent meta-analysis of prospective studies, functional magnetic resonance imaging (fMRI) markers of food cue reactivity might predict future eating and weight gain (Boswell and Kober, 2016).

Despite this progress, neuro-cognitive understanding of cue-driven overeating is hampered by the complicated and networked dynamics involved in reward processing and appetitive cue-reactivity (Haber, 2017; Hill-Bowen et al., 2021). Mesocorticolimbic regions often have reciprocal connections, with prefrontal areas responding to but also controlling ventral tegmental dopamine release (Lodge, 2011; Haber, 2017) and dense fronto-amygdalar and amygdalar-striatal connections involved in encoding and updating reward values (Janak and Tye, 2015). Furthermore, these regions receive and project both excitatory and inhibitory connections with reward and anti-reward effects (Lodge, 2011; Bouarab et al., 2019) and food cue-reactivity in obese individuals may engage both positive and negative reward processing, which are respectively involved in “wanting” and approach responses and guilt-associated avoidance (Carnell et al., 2012; Liu et al., 2019). fMRI food cue-reactivity studies often test for region- or voxel-wise blood oxygenation level-dependent (BOLD) signal differences within a general linear modeling (GLM) framework (Letra et al., 2017), which cannot clarify how observed activations or deactivations reflect food cue processing. This manuscript complements the literature by using dynamic causal modeling (DCM) to investigate the underlying neural dynamics of cue-reactivity in overweight and obese individuals with food-induced craving. DCM is a common framework for causal inference on effective connectivity and includes biologically plausible, explicit assumptions about the hidden neuronal state-space and its relationship to observed BOLD signal (Friston et al., 2003). Based on these assumptions, DCM involves the specification and comparison of models of task-related interaction among and within neural populations and the subsequent estimation of model parameters (Friston et al., 2003; Razi and Friston, 2016). This allows us to investigate reciprocal connections within and between regions of the reward network, tease apart inhibitory and excitatory connectivities, and assess the modulation of these interactions by food cues, shedding light on some of the neural dynamics which may underlie food cue-reactivity.

## Materials and Methods

### Participants

Overweight/obese volunteers who self-reported frequent food cravings were recruited via specialized nutrition clinics and through online advertisements and flyers. Frequent food craving was defined as reporting three or more episodes of food craving per day on average during the last month, and was assessed with the following question on the self-reported demographic questionnaire: “How many food cravings per day did you experience during the last month?”. Respondents needed to be 18–60 years old and were excluded if they: (1) reported a healthy BMI (19–25 kg/m2); (2) had a current or past history of psychiatric or neurological disorders; (3) had a current or past history of eating or gastrointestinal or substance use disorders, or metabolic or endocrine disease; (4) used current psychopharmacological medication; (5) were pregnant; (6) suffered from claustrophobia; (7) had any metallic implants; (8) reported having severe food allergies; (9) reported having special diets; or (10) had low food cue-induced craving.

Fifty-six candidates provided written informed consent and were screened for eligibility to enter the study. Two had low food cue responsiveness and were excluded from further assessment. Fifty-four participants fulfilled all inclusion/exclusion criteria, of whom four were excluded from the study—one for excessive head motion during scanning, 2 for unanticipated claustrophobia, and one due to an intracranial lesion. The final sample included in the analyses consisted of 50 overweight/obese volunteers aged 21–59 (*M* = 35.33, *SD* = 9.82; 34 female). Detailed demographic and clinical information of the participants is reported in Table 1. The study protocol was approved by the ethics committee of the Iran University of Medical Sciences (IR.IUMS.REC.1396.0459).

**TABLE 1:** Sample characteristics (*n* = 50).

Variable	Mean	*SD*
Age	35.33	9.82
Height (m)	1.67	0.10
Weight (kg)	82.63	14.16
BMI (kg m^–2^)	29.67	3.56
Education (Y)	16.94	2.38
CES	11.06	5.78
**DASS**	20.84	13.16
Depression (0–21)	6.16	5.21
Anxiety (0–21)	5.02	4.22
Stress (0–21)	9.67	5.14
**TFEQ**		
Hunger (0–30)	14.61	5.47
Cognitive restraint (0–12)	5.71	2.72
Emotional eating (0–6)	3.53	1.62
**EDDQ**		
Body image (0–24)	14.90	5.39
Overeating (0–8)	4.54	2.08
Compensatory behaviors (0–56)	3.10	3.98
**FCQ-Trait**		
Lack of control under environmental cues	29.37	15.04
Thoughts or preoccupation with food	13.04	9.17
Hedonic hunger	31.20	13.10
Emotions before or during food craving	11.24	5.70
Guilt from craving	7.20	4.50
**FCQ-State**	32.06	15.98
Intense desire to eat	7.66	3.96
Anticipation of positive reinforcement that may result from eating	7.74	3.93
Anticipation of relief from negative states and feelings as a result of eating	5.91	4.04
Obsessive preoccupation with food or lack of control over eating	6.38	4.20
Craving as a physiological state	4.36	3.89

*Values are reported as mean (SD). BMI, Body Mass Index; CES, Compulsive Eating Scale; DASS-21, Depression Anxiety Stress Scales-21; EDDQ, Eating Disorder Diagnostic Questionnaire; FCQ-T, Food Craving Questionnaire-Trait; FCQ-S, Food Craving Questionnaire-State; TFEQ-R18, Three-Factor Eating Questionnaire-R18.*

### Experimental Procedures

**FIGURE 1:**
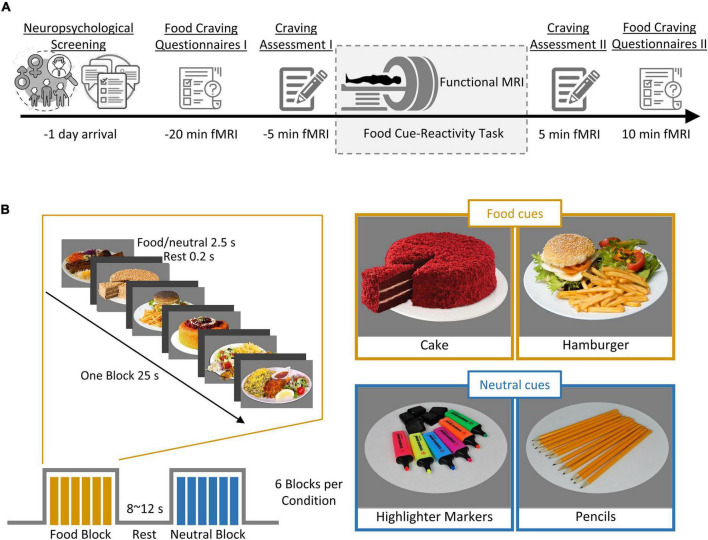
Outline of the experimental procedure and cue-reactivity paradigm. **(A)** Experimental procedure. First, individuals underwent screening for their neuropsychological functioning (measured by self-reported questionnaire; see Table 1 for details). Each participant (*n* = 50) then underwent a food cue-reactivity training task. On the experiment day, participants completed two food craving questionnaires: FCQ-state and FCQ-Trait and subsequently underwent an MR scan with the food cue-reactivity task. Immediately before and after the MR scan, participants rated their self-reported food craving, hunger, and affective states (anger, anxiety, awareness, drowsiness, happiness, sadness). **(B)** Food cue-reactivity paradigm. In the food cue-reactivity task, participants saw 12 blocks of 6 pictures each (six blocks with food cues and six blocks with neutral cues). Each block was followed by an inter-block interval with a duration between 8 and 12 s. In total, participants saw 72 cues over 342 s.

Figure 1 shows an overview of the experimental procedure. During the screening session, participants completed a battery of baseline assessments. These included a self-reported demographic questionnaire, the Depression Anxiety Stress Scales-21 (DASS-21; Sahebi et al., 2005), the Three-Factor Eating Questionnaire-R18 (TFEQ-R18; Mostafavi et al., 2017), the Eating Disorder Diagnostic Scale (EDDS; Stice et al., 2000), and the Compulsive Eating Scale (CES; Mostafavi et al., 2016). This was followed by familiarization with the cue-reactivity task during a training session in which participants were instructed to rate their momentary craving induced by several food cues on a 0–100 VAS scale and lasted ∼3 min. Average cue-induced craving from this session was used to exclude participants with low cue-induced food craving, defined as a mean cue-induced craving score below 80 out of 100. Participants who were eligible after this screening session were then scanned in one fMRI session.

On the magnetic resonance imaging (MRI) scan day, participants arrived in the morning between 8:30 and 10:30 am after a fasting period of at least 2 h. Participants were asked to fill out the Food Craving Questionnaire-Trait (FCQ-T; Kachooei and Ashrafi, 2016), as well as the Food Craving Questionnaire-State (FCQ-S; Cepeda-Benito et al., 2000). After ratings of feelings of the current craving, hunger, prospective consumption, food control, and affective states (anger, anxiety, awareness, drowsiness, happiness, and sadness) on a 0–100 VAS (Figure 1A, Craving Assessment I), participants began the food-cue reactivity paradigm (in the MRI scanner). After completion of the scans, participants again rated their feeling of craving, hunger, prospective consumption, food control, and affective states (Figure 1A, Craving Assessment II).

### Functional MRI Food-Cue Reactivity Paradigm

Stimuli were presented in a block design, with 12 blocks of 6 images each (six blocks with food cues and six blocks with neutral cues), where participants were asked to look at the cues and pay close attention. Each cue lasted 4000 ms. Each block was followed by a jittered inter-block interval with a duration between 8 and 12 s (Figure 1B). In total, participants viewed 72 cues over 342 s. The neutral cues originated from the Full4Health Image Collection (Charbonnier et al., 2016) and the food cues were taken from the Internet or in the course of the study. Neutral images were matched for content to the food images.

### MRI Data Acquisition

Functional MRI images were collected using a SIEMENS 3.0T scanner (MAGNETOM Prisma, SIEMENS, Germany) using a 20-channel head coil at the Iranian National Brain Mapping Laboratory. A total of 167 functional T2*-weighted images were acquired in an interleaved slide order with a voxel size of 3 × 3 × 3 mm, a repetition time (TR) = 2500 ms, echo time (TE) = 23 ms, and flip angle (FA) = 70°. Each volume consisted of 43 axial slices and a field of view (FOV) = 192 × 192 mm. Furthermore, we acquired a T1-weighted structural image (magnetization prepared rapid acquisition gradient echo, MPRAGE) with 176 slices, a TR = 1810 ms, TE = 3.45 ms, and a FA = 7°.

### Functional MRI Data Analysis

Functional MRI data were analyzed using MATLAB R2018b (Mathworks Inc., Natick, MA, United States) and SPM12 software (Wellcome Trust Centre for Neuroimaging, London, United Kingdom^1^). The fMRI scans were corrected for slice timing and head motion. The structural T1 image was co-registered to the mean functional image generated during realignment. The structural images were segmented into GM, WM, CSF, skull, soft-tissue, and air partitions following co-registration with the mean T2* image. The functional images were spatially normalized to the Montreal Neurological Institute (MNI) standardized space, and smoothed with a 6-mm full-width at half-maximum Gaussian kernel.

We used a general linear model (GLM) to obtain individual statistical parametric maps. At the single subject level, the experimental conditions (i.e., food and neutral) were defined by stimulus onset and the duration of each experimental trial. Experimental conditions were modeled as separate regressors and convolved with a canonical hemodynamic response function. Six head motion parameters and DVARS (Power et al., 2012) were added as regressors of no interest into the first-level model. A high pass-filter with a cut-off frequency of 1/128 Hz was used to remove low-frequency drifts (Glover et al., 2000).

For each subject, the differential contrasts directly comparing the food to the neutral conditions were then entered into second-level random-effects t-test models. The individual contrast maps thresholded at *p* < 0.05 (uncorrected) whereas group-level contrast maps were corrected for multiple comparisons at a family-wise error rate *P*_FWE_ < 0.05 using Gaussian random field theory (Worsley et al., 1996; Nichols and Hayasaka, 2003).

### Definition of Regions of Interest and Time-Series Extraction

We selected the regions of interest (ROIs) based on an initial mass-univariate SPM analysis, as is usual in the DCM literature, seeking the simplest possible functional wiring diagram that accounts for the results of the SPM analysis. Based on aforementioned food craving findings in humans and the role in the reward network central to salient cue processing, we focused on the VTA, the amygdala, and the OFC. We then extracted BOLD time-series from regions of interest (ROI), based on the peak activations induced by food craving from the whole-brain univariate analysis (contrast: food > neutral). Here, we used uncorrected contrast maps for subject-level activation analyses to ensure that the five ROIs (i.e., VTA, left amygdala, left OFC, right amygdala, right OFC) required in the DCM analysis could be identified in most participants.

### Dynamic Causal Modeling

Based on the extraction of the principal eigenvariate time-series in the predefined ROIs of the VTA, left amygdala, left OFC, right amygdala, and right OFC, adjusted for the F-contrast modeling the all food and neutral regressors, we subsequently applied bilinear DCM (Friston et al., 2003) with the stochastic option to model effective functional connectivity between these ROIs (Friston et al., 2011; Li et al., 2011; Daunizeau et al., 2012).

In the present study, we specified a “parent” DCM model – which contained all free parameters, with three regions (i.e., the VTA, left amygdala, left OFC, right amygdala, right OFC) for each subject (Zeidman et al., 2019a). In a parent DCM model, all possible connectivity parameters were freely estimated, that is, there were extrinsic forward and backward connections between the VTA and amygdala, between the VTA and OFC, between the amygdala and OFC, between the left amygdala and right amygdala, between the left OFC and right OFC, as well as intrinsic self-connections of the regions (fixed connections, A matrix), and also all self-connections were allowed to be modulated by food stimuli (contextual modulation, B matrix). Limiting modulatory effects to the self-connections enables biological interpretability and generally facilitates a more identifiable model (Zeidman et al., 2019a). The external inputs (food and neutral stimuli) were exerted on the nodes as driving input (exogenous inputs, C matrix). This parent DCM model was estimated for each subject using Bayesian model inversion (Friston et al., 2015).

To estimate group-level parameters in the craving network, we performed a second-level Parametric Empirical Bayes (PEB) analysis (Friston et al., 2015, 2016). We used a linear PEB analysis of the parent model estimated for all subjects. After estimating the parameters of the parent PEB model, we performed a Bayesian model reduction (BMR) to prune away any insignificant connectivity parameters from the parent model until the model evidence was not improved (Friston et al., 2016; Zeidman et al., 2019b). In brief, this procedure is known as a greedy search over the model space to optimize model evidence. We here used the BMR as a *post hoc* model selection with only minimal constraints and performed an automatic search over nested PEB models. In the present study, significant connectivity parameters were determined with a posterior probability of *P* > 0.99.

### Group Factor Analysis

We used the group factor analysis (GFA) to identify latent variables that describe relationships between groups of variables with a sparsity constraint (Klami et al., 2015). GFA employs a sparse Bayesian estimation to identify latent factors that either explain away group-specific variation or describe a robust relationship between groups. Three variable groups were defined: (i) effective connectivity parameters; (ii) behavioral measures; and (iii) demographic measures. In total, the model included 23 connectivity parameters (with a posterior probability of *P* > 0.99), 12 behavioral measures (changes in self-reported craving and hunger, FCQ-State subscales (Supplementary Figure 1) *Lack of control*, *Desire*, *Positive reinforcement*, *Negative reinforcement*, *Physiological hunger*, and FCQ-Trait subscales (Supplementary Figure 1) *Lack of control*, *Emotions*, *Guilt*, *Hunger*, *Thoughts*), and three self-report measures (age, BMI, education). Variables were *z*-normalized to have zero mean and unit variance, a form suitable for GFA. To minimize the risk of identifying spurious latent factors, sparse Bayesian estimation was repeated 10 times and only factors that were robust across 10 replicates of the GFA were extracted (Peng et al., 2021; White et al., 2021).

As a less robust, complementary test for neuro-behavioral associations, we used Pearson’s correlation tests to assess bivariate associations between estimated neural parameters and behavioral variables. All Pearson’s correlations and group factor analysis were conducted in statistical software R version 4.0.5, and the group factor analysis was calculated using the function *gfa* in R package GFA (Leppäaho et al., 2017; R Core Team, 2020).

## Results

### Behavioral Data

**FIGURE 2:**
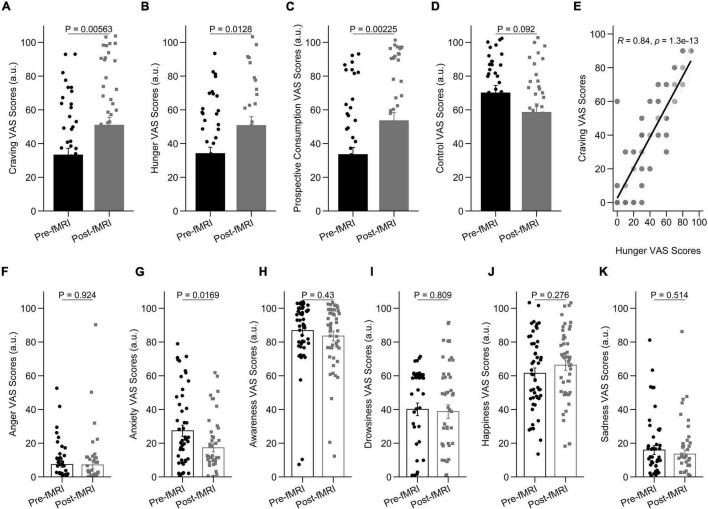
Behavioral results in the cue-reactivity condition. **(A–D)** Representative bar charts showing main effect of cue-reactivity on **(A)** craving; **(B)** hunger; **(C)** prospective consumption; and **(D)** food control before and after food cue-reactivity task. **(E)** Participants’ scores on the self-reported craving and hunger were significantly positively correlated (*R* = 0.84; *P* < 0.001; Pearson’s correlation). **(F–K)** Changes in self-reported score of affective states [anger **(F)**, anxiety **(G)**, awareness **(H)**, drowsiness **(I)**, happiness **(J)**, and sadness **(K)**] before and after food cue-reactivity task. Data in bar charts are represented as mean ± SEM.

To assess behavioral cue-reactivity effects on craving, hunger, prospective consumption, and food control ratings, we compared data directly before and after the fMRI session. After the fMRI session, participants reported increased craving [*t*(89.98) = –2.84, *P* = 0.005], hunger [*t*(54.89) = –2.57, *P* = 0.012], prospective consumption [*t*(93.13) = –3.14, *P* = 0.002], and decreased food control [*t*(63.19) = 1.71, *P* = 0.092] compared with before they went into the scanner (Figures 2A–D). In agreement with this observation, hunger VAS scores were directly correlated with food craving scores, such that individuals with higher craving ratings demonstrated stronger increase of hunger scores following cue-reactivity paradigm (*R* = 0.84; *P* < 0.001; Pearson’s correlation) (Figure 2E).

As expected, after the fMRI session, participants did not report a statistically significant change in anger [*t*(86.18) = 0.096, *P* = 0.924; Figure 2F], awareness [*t*(94.83) = 0.79, *P* = 0.43; Figure 2H], drowsiness [*t*(94.15) = 0.24, *P* = 0.809; Figure 2I], sadness [*t*(94.94) = 0.66, *P* = 0.514; Figure 2K], and happiness [*t*(94.95) = –1.095, *P* = 0.276; Figure 2J] compared with before they went into the scanner. There was however a significant decrease in anxiety state following cue-reactivity paradigm [*t*(87.40) = 2.43, *P* = 0.016] (Figure 2G).

### General Linear Modeling Analysis of Food Cue Reactivity and Regions of Interest Selection

**FIGURE 3:**
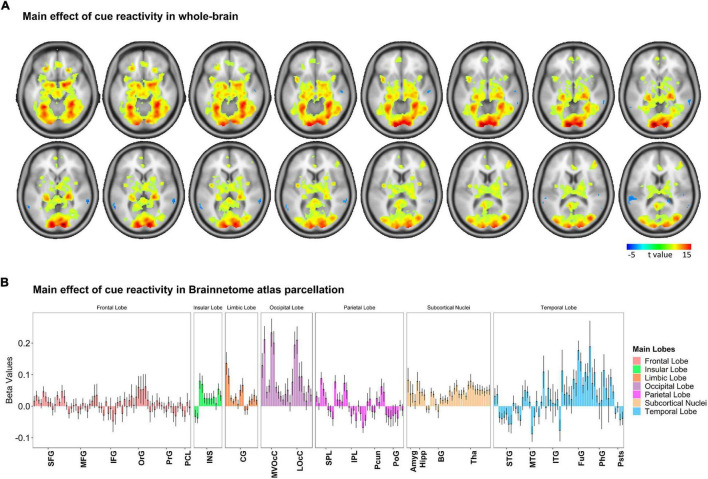
Whole-brain response to the task-based fMRI in contrasts of Food > Neutral. **(A)** Brain activation maps and **(B)** changes in brain activation in Brainnetome (BNA) regions. Based on the whole-brain analysis, changes in brain activation (contrast: food > neutral) in terms of beta values obtained from the general linear modeling (GLM) are represented to examine how food cue-reactivity influenced the brain’s circuitry. The details of brain activation changes for each subregion of the Brainnetome Atlas are shown in Supplementary Figure 2, and the ontology and nomenclature of brain areas and their abbreviations in the Brainnetome Atlas and associated Brodmann areas are listed in Supplementary Table 1. Data in bar charts are represented as mean ± SEM. SFG, superior frontal gyrus; MFG, middle frontal gyrus; IFG, inferior frontal gyrus; OrG, orbital gyrus; PrG, precentral gyrus; PCL, paracentral lobule; STG, superior temporal gyrus; MTG, middle temporal gyrus; ITG, inferior temporal gyrus; FuG, fusiform gyrus; PhG, parahippocampal gyrus; pSTS, posterior superior temporal sulcus; SPL, superior parietal lobule; IPL, inferior parietal lobule; Pcun, precuneus; PoG, postcentral gyrus; INS, insular gyrus; CG, cingulate gyrus; MVOcC, medioventral occipital cortex; LOcC, lateral occipital cortex; Amyg, amygdala; Hipp, hippocampus; BG, basal ganglia; Tha, thalamus.

To examine how cue-reactivity influenced the brain’s mesocorticolimbic circuitry, we analyzed BOLD activity measured during the food cue-reactivity task (contrast: food > neutral) using a random-effects model. Functional MRI findings were reported at the whole-brain level and within Brainnetome atlas regions (Figure 3 and Supplementary Figure 2, *p* < 0.05 FWE corrected). Investigating BOLD responses to food compared with neutral cues yielded highly significant activations in the amygdala, parahippocampus, insula, visual and orbitofrontal cortex (Figure 3A). Similar to previous observations, we found three peaks in the left mesocorticolimbic reward pathway [VTA (peak at MNI coordinate: –2, –14, –16), amygdala (peak at MNI coordinate: –18, –4, –16), OFC (Brodmann’s area 11; peak at MNI coordinate: –24, 30, –18)] and two peaks in the right mesocorticolimbic reward pathway [amygdala (peak at MNI coordinate: 18, –4, –16), OFC (Brodmann’s area 11; peak at MNI coordinate: 24, 30, –18)]. For each of those peak locations, we created an ROI (a sphere of 6 mm radius around the peak) to be used in the PEB-DCM analysis.

### Effective Connectivity Network of Food Cue Reactivity

**FIGURE 4:**
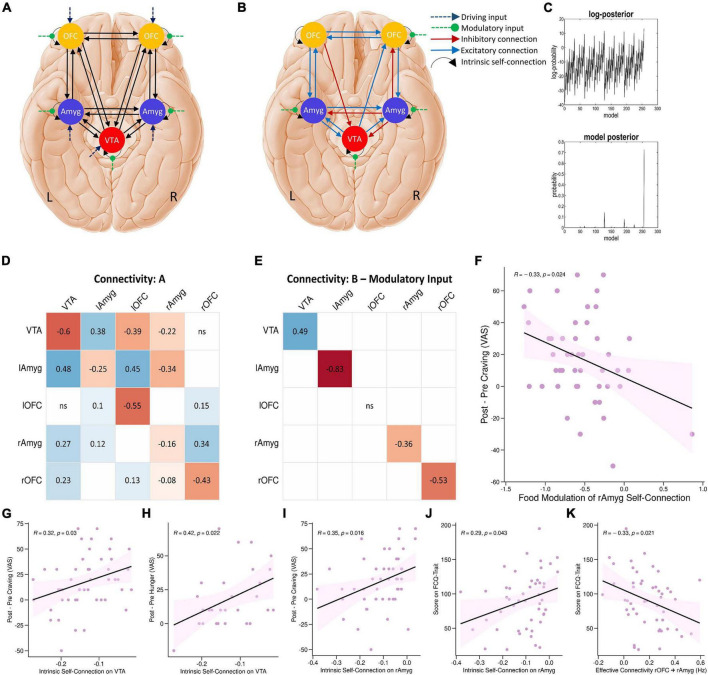
DCM results. **(A)** The parent DCM model was defined and inverted at the single-subject level, which included all intrinsic self-connections and extrinsic between-region connections in each hemisphere, food and neutral stimuli as driving input, and food modulation of all intrinsic connections. **(B)** The winning DCM model after *post hoc* Bayesian model selection, green arrows indicate modulation by the food stimuli. **(C)** The upper panel shows the range of log-posterior probabilities of all possible models examined. The bottom panel shows the posterior probability for all reduced models. It shows that the winning model had the posterior probability of 0.73, which indicates that the model had more evidence than any other model. The next most probable model was M128 with a posterior probability of 0.14 (the log-probability of 11.66). **(D,E)** The corresponding connectivity matrices for winning DCM model. Matrices A and B correspond to connection strengths and input modulations of connections, respectively. **(F)** Correlations between neural and behavioral findings. Individual parameter estimates of food modulation on the inhibitory intrinsic connection of the right amygdala correlated with craving changes (Δ craving effect post-fMRI - pre-fMRI) (*R* = –0.33; *P* = 0.024; Pearson’s correlation). Individual intrinsic self-connection of the VTA node predicted participants’ scores on the self-reported craving (*R* = 0.32; *P* = 0.03; Pearson’s correlation) **(G)** and hunger (*R* = 0.42; *P* = 0.022; Pearson’s correlation) **(H)**. Also, intrinsic self-connection of the right amygdala node correlated with participants’ scores on the self-reported craving (*R* = 0.35; *P* = 0.016; Pearson’s correlation) **(I)** and the FCQ-Trait (*R* = 0.29; *P* = 0.043; Pearson’s correlation) **(J)**. Individual extrinsic connections from the right OFC to right amygdala correlated with scores on the FCQ-Trait (*R* = –0.33; *P* = 0.021; Pearson’s correlation) **(K)**. lAmyg, Left Amygdala; lOFC, Left Orbitofrontal Cortex; rAmyg, Right Amygdala; rOFC, Right Orbitofrontal Cortex; VTA, Ventral Tegmental Area.

To investigate the network mechanisms underlying the food cue-reactivity effects, we next employed DCM to infer effective connectivity of craving-related regions during the food cue-reactivity task. We selected five regions revealed by the food > neutral contrast: the left amygdala, left OFC, right amygdala, right OFC—which are all frequently implicated in the reward processing—and the VTA—to model the driving input (Figure 4A).

*Post hoc* Bayesian model selection (Friston et al., 2016) identified the model with the best evidence by comparing the evidence over nested PEB models. This resulted in an effective neural network with reciprocal positive connections between the left amygdala and left OFC, between the left amygdala and VTA, and between the left OFC and right OFC, positive connections from the VTA to right amygdala, from right OFC to right amygdala, from the VTA to right OFC, and from left amygdala to right amygdala, as well as negative connections from left OFC to VTA, from right amygdala to VTA and right OFC, and from right amygdala to left amygdala. There were also inhibitory self-connections in the VTA, right OFC, right amygdala, and left amygdala nodes. More interestingly, the model also included negative modulation by food stimuli of the self-connection of the right OFC, right amygdala, and left amygdala regions, as well as positive modulation of the self-connection of the VTA region by food stimuli (Figures 4B–E and Supplementary Table 2).

### Relationships Between Neural, Behavioral and Demographic Variables

**FIGURE 5:**
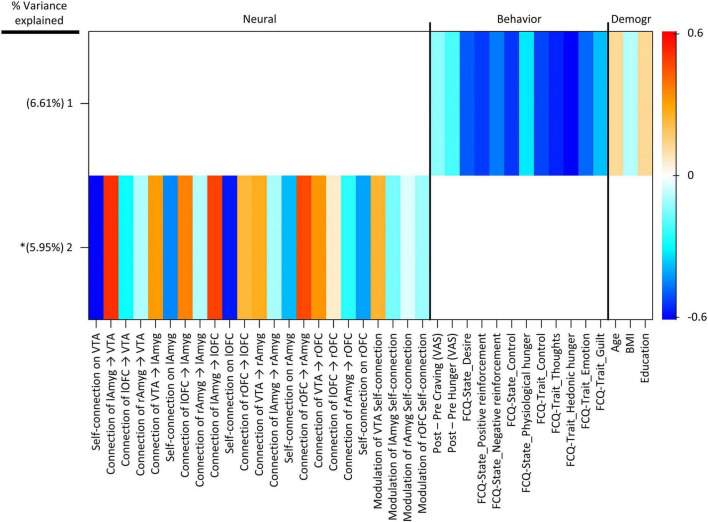
Group factor analysis (GFA) robust factor loadings. Heatmap colors indicate the weight of each group variable loading. Robust group factors are sorted in descending order by mean percent variance explained across all groups. Asterisks indicate latent variable that contained at least one latent variable loading whose 95% credible interval did not contain 0. Latent variable labels, indicating the variable group onto which each latent variable was loaded and the order by most variance explained, are given in the upper key beneath the heatmap. BMI, Body Mass Index; Demogr, Demographic; FCQ-State, Food Craving Questionnaire-State; FCQ-Trait, Food Craving Questionnaire-Trait; lAmyg, Left Amygdala; lOFC, Left Orbitofrontal Cortex; rAmyg, Right Amygdala; rOFC, Right Orbitofrontal Cortex; VTA, Ventral Tegmental Area.

Group factor analysis extracted two robust latent variables (Figure 5), together explaining approximately 12.56% of the variance across variable groups, no robust cross-unit latent factors were identified between the neural group and both behavioral and demographic variable groups. In other words, the GFA failed to show any coherence between the neural group and both behavioral and demographic variable groups in latent variable space. In contrast, the GFA identified a robust latent factor that loaded across behavioral and demographic units of analysis. However, the mean-variance explained was 20.76 and 1.23% within the behavioral and demographic variable groups, respectively. Thus, while this latent factor technically contained loadings across behavioral and demographic levels of analysis, the behavioral variables accounted for virtually all of the variance explained.

Finally, we tested for bivariate correlations between behavioral variables and estimated excitatory/inhibitory coupling and modulatory neural parameters. These Pearson’s correlation tests included individual estimated parameters from the winning PEB and either cue-induced food craving or hunger (defined as changes in food craving and hunger, post-fMRI – pre-fMRI) or FCQ-trait and FCQ-state scores. These correlation tests revealed a significant positive correlation (*R* = –0.33; *P* = 0.024, *n* = 50, Pearson’s correlation) indicating that negative modulation of the inhibitory intrinsic connection of the right amygdala by food cues is correlated with cue-induced increases in food craving (Figure 4F). There were other significant correlations between behavioral variables and inhibitory self-connections in the VTA and right amygdala nodes and the extrinsic connectivity from the right OFC to right amygdala, from the VTA to left amygdala, and from the right amygdala to right OFC (Figures 4G–K and Supplementary Figure 3).

## Discussion

This study adds to the scant literature using causal inference to investigate neural food cue-reactivity, selecting regions of the reward network with differential food > neutral activation before inferring the neural interactions likely to underly the observed BOLD signal. Among causal inference frameworks, DCM is uniquely tailored for fMRI and has several advantages (Bielczyk et al., 2019): DCM explicitly models the task-related hemodynamic response and enables inference on neural level (Razi and Friston, 2016), and it allows for the specification and comparison of sophisticated models with modulated excitatory and inhibitory connections (Bielczyk et al., 2019). Furthermore, empirical investigations generally support DCM’s validity (Papadopoulou et al., 2015) and reliability (Bernal-Casas et al., 2013; Almgren et al., 2018). We are aware of two studies utilizing DCM to investigate neural food cue processing: Tiedemann et al. (2017) demonstrated that central insulin inhibits the excitatory VTA-nucleus accumbens connection in fasted insulin-sensitive (but not insulin-resistant) individuals and that this modulatory effect is correlated with lower food valuation following insulin administration; and He et al. (2019) utilized a mixed cue-reactivity/go-nogo task to elucidate the network interactions underlying response inhibition during exposure to food cues, showing that interactions are influenced by food deprivation and that this influence is associated with BMI among college students. Subsequently, the present investigation is the first to focus purely on the neural dynamics engaged by a cue-reactivity task and their modulation by food cues in a moderately sizeable sample of overweight or obese individuals (*n* = 50), and the relationship between these neural dynamics and variables such as BMI, state and trait food craving, and cue-induced hunger and craving. In addition, this investigation is the first food cue-reactivity study to use a sparse Bayesian GFA to assess the latent factors that describe relationships between the neural and behavioral variables, as well as exploratory neuro-behavioral correlation tests. Furthermore, this study included only participants with cue-induced food craving, excluding individuals whose overweight or obese status is less likely caused by cued overeating.

### Ventral Tegmental Area, Dopaminergic Center of the Reward Network

Among regions of the reward network (Haber, 2017), the VTA, bilateral OFCs and amygdalae showed significant food cue-reactivity. DCM results suggest that these activations result mostly from reciprocal excitatory and inhibitory connections across the network, with food cues disinhibiting the left OFC, bilateral amygdalae, and the VTA. These regions have been consistently implicated in food cue-reactivity studies in overweight and obese individuals, and their reactivity to appetitive food cues can predict overeating and body composition changes (Val-Laillet et al., 2015; Boswell and Kober, 2016; Letra et al., 2017; Devoto et al., 2018). The VTA contains a multitude of excitatory and inhibitory neural populations which synapse mainly at the ventral striatum and corticolimbic regions, including the prefrontal cortex and the amygdala (Trutti et al., 2019). The region’s hyper-activation in response to food cues is a key part of incentive sensitization theories of obesity (Robinson et al., 2016; Stice and Burger, 2019): These theories generally predict that as appetitive conditioning to food-relevant cues develops, exposure to such cues would come to activate the VTA, which has excitatory dopaminergic connections to other regions of the reward network (Morales and Margolis, 2017; Devoto et al., 2018). Furthermore, there is evidence that this activation partly results from the relief of tonic self-inhibition which is maintained by anti-reward GABAergic neurons in the VTA (Jalabert et al., 2011; van Zessen et al., 2012; Bouarab et al., 2019). This is in line with our observations that food cues negatively modulate this self-connection and disinhibit the VTA, and the clinical relevance of tonic VTA inhibition might be highlighted by our exploratory observation of negative correlations between the strength of intrinsic inhibition and both cue-induced hunger and food craving.

### Amygdala, Encoding Valence and Salience in Reward Learning

The amygdala seems particularly central in the food cue-reactivity network based on DCM results, with reciprocal connections between both amygdalae and the VTA and OFC ipsilaterally. Previous food cue-reactivity research has demonstrated that amygdalar cue-reactivity is associated with BMI, compulsive overeating and symptoms of food addiction (Filbey et al., 2012; Pursey et al., 2019; Stice and Burger, 2019), suggesting that the amygdala is particularly involved in overweight and obesity driven by cued over-eating. This could be explained by an incentive sensitization theory of obesity, since amygdalar circuits are essential for reward valuation and associate stimuli to reward valence to guide behavior in conjunction with the prefrontal cortex (Murray, 2007; Lichtenberg et al., 2017; Devoto et al., 2018). This valence encoding may explain the bivariate correlation between food-mediated amygdalar disinhibition and induced craving, consistent with evidence that amygdalar cue-reactivity encodes subjective food cue valuation and predicts food consumption (Gottfried et al., 2003; Tiedemann et al., 2020). Another possibility is that the observed disinhibition is mediated by negative valuation of food cues: The amygdalae encode both the negative and positive reward valence of conditioned stimuli (Murray, 2007; Janak and Tye, 2015), and might be involved in compulsive overeating despite and because of negative associations and feelings of guilt induced by food cues (Moore et al., 2017). However, this is less likely to be the primary mechanism since we observed no evidence of changes in negative affect after the scan.

Another observation is the striking lateralization of effective amygdalar connectivity: Only the right amygdala is disinhibited by food cues and receives excitatory connections from the VTA, the contralateral amygdala, and the ipsilateral OFC, an effective connectivity pattern which would shift activity from across the reward network to the right amygdala during food cue-reactivity. This striking hemispheric asymmetry would not have been observable in the conventional GLM analysis, and exploratory neuro-behavioral correlation tests suggest that it should not be overlooked: in the right but not the left amygdala, both weaker self-inhibition and stronger food-induced disinhibition are correlated with a higher trait food craving and greater cue-induced craving. This accords with some evidence that right amygdalar volume may be associated with BMI in overweight individuals (Orsi et al., 2011), and meta-analytical evidence that both drug and food cues activate the right amygdala (García-García et al., 2014). Multiple hypotheses have been put forward to explain observations of amygdalar asymmetry in affective neuroscience, including suggestions that the right amygdala may be more sensitive to pictorial cues (Markowitsch, 1998) and responds and habituates more quickly to emotional stimuli (Sergerie et al., 2008); or that the right amygdala is engaged more strongly by negative reward and valence, while the left amygdala is more involved in positive reward and valence processing (Lee et al., 2004). While neuro-behavioral correlations suggest that the right amygdala may be uniquely relevant to food craving in overweight and obese individuals, more extensive research is required to clarify the reproducibility and implications of the observed functional lateralization of the amygdalae, especially given its multifaceted role in cue-reactivity in obesity.

### Orbitofrontal Cortex, Integrator of Sensation, Reward and Behavior

The OFC likely has a sophisticated involvement in food cue-reactivity, receiving food-relevant sensory inputs and interacting with the reward network to dynamically associate cues and reward expectations with decision-making (Wang et al., 2004; Havermans, 2013; Haber, 2017). In accordance with previous observations, our model includes both excitatory connections from the VTA to the OFC and inhibitory projections from the OFC to the VTA (Lodge, 2011; Seabrook and Borgland, 2020). We also observed reciprocal OFC-amygdalar connections and a negative correlation between trait craving and right OFC-amygdala connectivity, which is notable since the two regions communicate extensively through the uncinate fasciculus (Bracht et al., 2009) and their cue-related communication may be impaired in obesity (Stoeckel et al., 2009). Substantial research has implicated this reciprocal interaction in value-based decision-making broadly, with amygdalar projections to the OFC mediating cue-associated reward expectancies and the OFC modulating amygdalar cue-reactivity to link reward expectancy and behavior (Tromp et al., 2012; Lichtenberg et al., 2017; Seabrook and Borgland, 2020). A final noteworthy observation is that while the right and left OFC are similarly activated by food cues, their effective connections are asymmetrical: the right OFC was disinhibited by food cues and received an excitatory connection from the VTA, while the left OFC sent an inhibitory connection to the VTA and was not directly affected by food cues. Furthermore, only the right OFC received an inhibitory amygdalar connection. This might suggest a tonic regulatory role for the left OFC and a more dynamic food cue response in the right OFC, but hemispheric lateralization has also been observed in attentional, inhibitory and other neurocognitive processes potentially relevant to food cue-reactivity (D’Alberto et al., 2017; Lopez-Persem et al., 2020), and more research is required to clarify its implications.

### Limitations

Several limitations should be noted in the investigation. The present study did not include a control group (i.e., lean human subjects), so the specificity of observed neural patterns in overweight and obese individuals is unclear. Furthermore, we only examined appetizing food cues and since possible differences between appetizing and non-appetizing foods in neuronal food processing have previously been observed (Nummenmaa et al., 2012), our results should be generalized with caution.

## Conclusion

This work adds to the growing literature on the neural network dynamics and effective connections which underlie food cue-reactivity in overweight and obese individuals, and uses DCM to select a plausible model of reward network function during food cue-reactivity. Most regions of the reward network are disinhibited during exposure to food cues in the winning model, and activation largely converges on the right amygdala based on the balance of excitatory and inhibitory connections in the network. This model largely accords with the incentive sensitization theory of obesity, which would account for the activation of regions involved in encoding reward valence and excitatory outgoing signals from the VTA in response to food cues (Robinson et al., 2016; Devoto et al., 2018; Stice and Burger, 2019). Furthermore, our results complement recent evidence that neural food cue-reactivity predicts overeating and BMI (Boswell and Kober, 2016) and that reward network functional connectivity may explain the therapeutic impact of interventions (Makaronidis and Batterham, 2018; Kerem et al., 2020), with exploratory tests identifying significant correlations between lower intrinsic inhibitory tone in the VTA and right amygdala and right amygdalar disinhibition during food cue-reactivity on the one hand and food-induced craving and hunger on the other; however, it should be noted that the more robust GFA extracted no common latent factors between neural and behavioral variables, similar to other recent work suggesting that such cross-modal relationships may be difficult to identify using current paradigms (Peng et al., 2021). Our work points to potential avenues for interventions targeting the reward network; in particular at the VTA, right amygdala, and the right OFC-amygdala connection. Moving forward, prospective and interventional studies are required to further elucidate the reward network dynamics with etiologic relevance to obesity and establish the clinical relevance of network parameters.

## Data Availability Statement

The raw data supporting the conclusions of this article will be made available by the authors, without undue reservation.

## Ethics Statement

The studies involving human participants were reviewed and approved by the Ethics Committee of Research, Iran University of Medical Science (IR.IUMS.REC.1396.0459). The patients/participants provided their written informed consent to participate in this study.

## Author Contributions

PG-A, AS, and HE designed the research. PG-A and AS performed the research and wrote the manuscript. PG-A analyzed the data. HE and RMK reviewed and edited the manuscript and supervised the study. All authors approved the final manuscript.

## Conflict of Interest

The authors declare that the research was conducted in the absence of any commercial or financial relationships that could be construed as a potential conflict of interest.

## Publisher’s Note

All claims expressed in this article are solely those of the authors and do not necessarily represent those of their affiliated organizations, or those of the publisher, the editors and the reviewers. Any product that may be evaluated in this article, or claim that may be made by its manufacturer, is not guaranteed or endorsed by the publisher.

## Footnotes

1
http://www.fil.ion.ucl.ac.uk/spm/
