# Supplementary material for: Task-Dependent Effective Connectivity of the Reward Network During Food Cue-Reactivity: A Dynamic Causal Modeling Investigation
Source: Front Behav Neurosci. 2022 Jun 24;16:899605. doi: 10.3389/fnbeh.2022.899605 (PMC9263922; doi:10.3389/fnbeh.2022.899605)
Supplement: Supplementary file 1 [file Image_1.pdf]

SUPPLEMENTARY MATERIAL

<u>Title:</u> Task-Dependent Effective Connectivity of the Reward Network During Food Cue-Reactivity: A Dynamic Causal Modelling Investigation

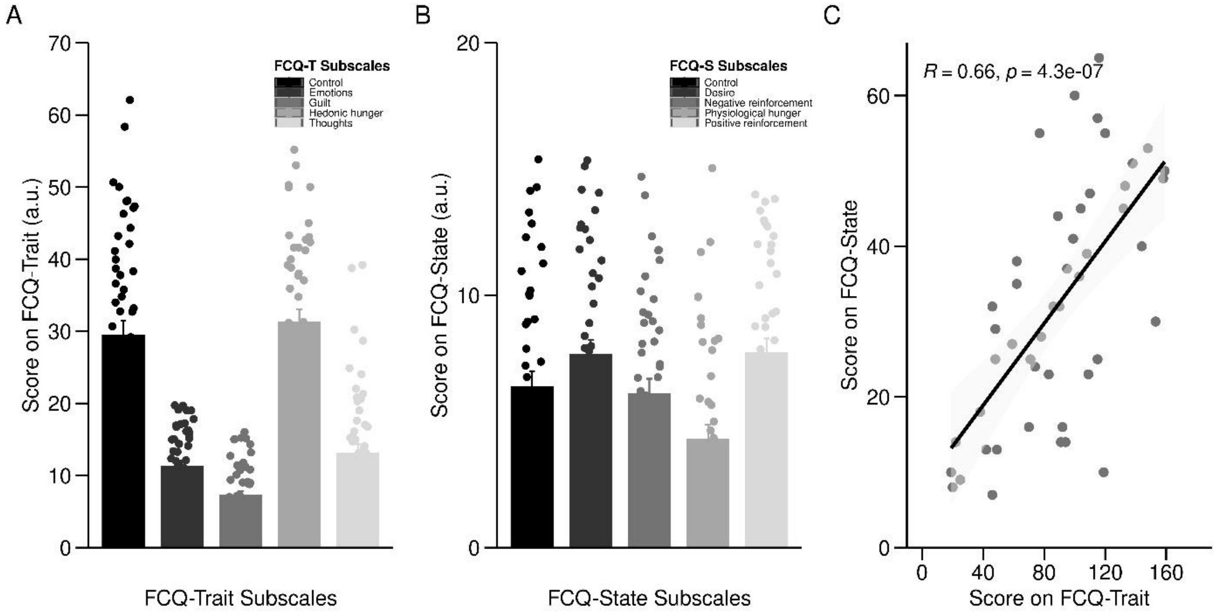

Supplementary Figure 1 | Behavioral signature of the FCQ-Trait and –State. (A) Mean (±s.e.m.) craving ratings on the FCQ-Trait subscales *Lack of control*, *Emotions*, *Guilt*, *Hunger*, *Thoughts* before participants went into the scanner. **(B)** Mean (±s.e.m.) craving ratings on the FCQ-State subscales *Lack of control*, *Desire*, *Positive reinforcement*, *Negative reinforcement*, *Physiological hunger* before participants went into the scanner. **(C)** Participants' scores on the FCQ-Trait and -State were significantly positively correlated.
