# Supplementary material for: Task-Dependent Effective Connectivity of the Reward Network During Food Cue-Reactivity: A Dynamic Causal Modeling Investigation
Source: Front Behav Neurosci. 2022 Jun 24;16:899605. doi: 10.3389/fnbeh.2022.899605 (PMC9263922; doi:10.3389/fnbeh.2022.899605)
Supplement: Supplementary file 2 [file Image_2.pdf]

SUPPLEMENTARY MATERIAL

Title: Task-Dependent Effective Connectivity of the Reward Network During Food Cue-Reactivity: A Dynamic Causal Modelling Investigation

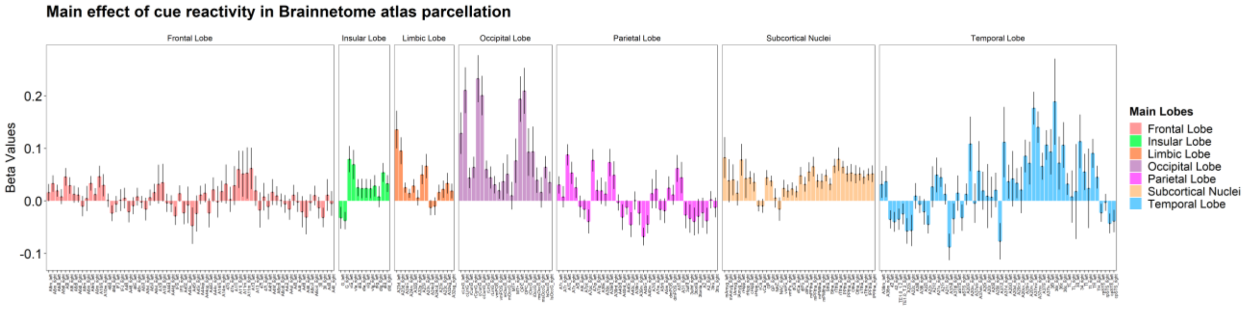

Supplementary Figure 2 | Whole-brain response to the task-based fMRI in Brainnetome atlas (BNA) parcellation. In an exploratory approach, brain activation during the food cue-reactivity task was extracted in fMRI data collection. Based on whole-brain analysis, changes in brain activation in terms of beta values obtained from the general linear modeling (GLM) are represented for Brainnetome (BNA) regions. Bars show mean value and error bars show standard error of the beta values across the population.
