# Supplementary material for: Task-Dependent Effective Connectivity of the Reward Network During Food Cue-Reactivity: A Dynamic Causal Modeling Investigation
Source: Front Behav Neurosci. 2022 Jun 24;16:899605. doi: 10.3389/fnbeh.2022.899605 (PMC9263922; doi:10.3389/fnbeh.2022.899605)
Supplement: Supplementary file 3 [file Image_3.pdf]

SUPPLEMENTARY MATERIAL

<u>Title:</u> Task-Dependent Effective Connectivity of the Reward Network During Food Cue-Reactivity: A Dynamic Causal Modelling Investigation

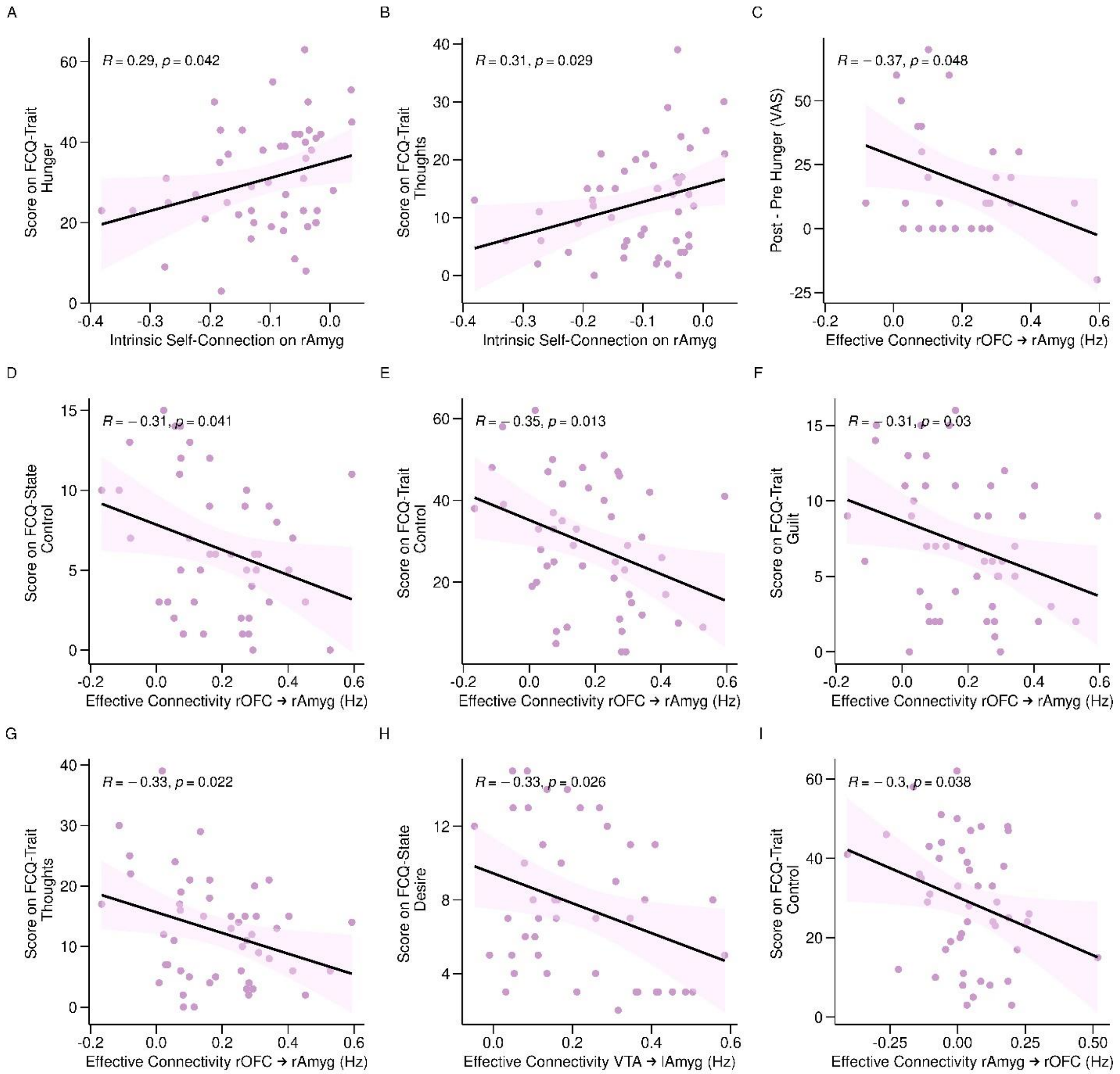

Supplementary Figure 3 | Correlations between neural and behavioral findings. Individual intrinsic self-connection of the right amygdala node correlated with participants' scores on the FCQ-Trait subscales *hunger* **(A)** and *thoughts* **(B)**. Individual extrinsic connection from the right OFC to right amygdala correlated with participants' scores on the self-reported hunger **(C)**, the FCQ-State subscale *control* **(D)**, and the FCQ-Trait subscales *control* **(E)**, *guilt* **(F)**, and *thoughts* **(G)**. Individual extrinsic connections from the VTA to left amygdala and from the right amygdala to right OFC correlated with the FCQ-State subscale *desire* **(H)** and the FCQ-Trait subscale *control* **(I)**. lAmyg, Left Amygdala; lOFC, Left Orbitofrontal Cortex; rAmyg, Right Amygdala; rOFC, Right Orbitofrontal Cortex; VTA, Ventral Tegmental Area.
