# Supplementary material for: Task-Dependent Effective Connectivity of the Reward Network During Food Cue-Reactivity: A Dynamic Causal Modeling Investigation
Source: Front Behav Neurosci. 2022 Jun 24;16:899605. doi: 10.3389/fnbeh.2022.899605 (PMC9263922; doi:10.3389/fnbeh.2022.899605)
Supplement: Supplementary file 4 [file Table_1.docx]

**SUPPLEMENTARY MATERIAL**

**Title:** Task-Dependent Effective Connectivity of the Reward Network During Food Cue-Reactivity: A Dynamic Causal Modelling Investigation

| **Supplementary Table 1** **\|** Ontology and nomenclature of brain areas in the Brainnetome Atlas. | | | | | | |
| --- | --- | --- | --- | --- | --- | --- |
| **Lobe** | **Gyrus** | **Modified cytoarchitectonic** | **Left hemisphere MNI (X, Y, Z)** | **Brodmann area** | **Right hemisphere MNI (X, Y, Z)** | **Brodmann area** |
| Frontal lobe | SFG, Superior frontal gyrus | A8m, medial area 8 | −5 ,15, 54 | 6 | 7, 16, 54 | 6 |
|  |  | A8dl, dorsolateral area 8 | −18, 24, 53 | 6 | 22, 26, 51 | 8 |
|  |  | A9l, lateral area 9 | −11, 49, 40 | 9 | 13, 48, 40 | 9 |
|  |  | A6dl, dorsolateral area 6 | −18, −1, 65 | 6 | 20, 4, 64 | 6 |
|  |  | A6m, medial area 6 | −6, −5, 58 | 6 | 7, −4, 60 | 6 |
|  |  | A9m, medial area 9 | −5, 36, 38 | 8 | 6, 38, 35 | 8 |
|  |  | A10m, medial area 10 | −8, 56, 15 | 10 | 8, 58, 13 | 10 |
|  | MFG, Middle frontal gyrus | A9/46d, dorsal area 9/46 | −27, 43, 31 | 10 | 30, 37, 36 | 9 |
|  |  | IFJ, inferior frontal junction | −42, 13, 36 | 8 | 42, 11, 39 | 8 |
|  |  | A46, area 46 | −28, 56, 12 | 10 | 28, 55, 17 | 10 |
|  |  | A9/46v, ventral area 9/46 | −41, 41, 16 | 46 | 42, 44, 14 | 10 |
|  |  | A8vl, ventrolateral area 8 | −33, 23, 45 | 8 | 42, 27, 39 | 9 |
|  |  | A6vl, ventrolateral area 6 | −32, 4, 55 | 6 | 34, 8, 54 | 6 |
|  |  | A10l, lateral area 10 | −26, 60, −6 | 10 | 25, 61, −4 | 10 |
|  | IFG, Inferior frontal gyrus | A44d, dorsal area 44 | −46, 13, 24 | 44 | 45, 16, 25 | 44 |
|  |  | IFS, inferior frontal sulcus | −47, 32, 14 | 46 | 48, 35, 13 | 46 |
|  |  | A45c, caudal area 45 | −53, 23, 11 | 45 | 54, 24, 12 | 45 |
|  |  | A45r, rostral area 45 | −49, 36, −3 | 47 | 51, 36, −1 | 45 |
|  |  | A44op, opercular area 44 | −39, 23, 4 | 45 | 42, 22, 3 | 45 |
|  |  | A44v, ventral area 44 | −52, 13, 6 | 44 | 54, 14, 11 | 44 |
|  | OrG, Orbital gyrus | A14m, medial area 14 | −7, 54, −7 | 10 | 6, 47, −7 | 10 |
|  |  | A12/47o, orbital area 12/47 | −36, 33, −16 | 47 | 40, 39, −14 | 47 |
|  |  | A11l, lateral area 11 | −23, 38, −18 | 11 | 23, 36, −18 | 11 |
|  |  | A11m, medial area 11 | −6, 52, −19 | 11 | 6, 57, −16 | 11 |
|  |  | A13, area 13 | −10, 18, −19 | 11 | 9, 20, −19 | 11 |
|  |  | A12/47l, lateral area 12/47 | −41, 32, −9 | 47 | 42, 31, −9 | 47 |
|  | PrG, Precentral gyrus | A4hf, area 4(head and face region) | −49, −8, 39 | 6 | 55, −2, 33 | 6 |
|  |  | A6cdl, caudal dorsolateral area 6 | −32, −9, 58 | 6 | 33, −7, 57 | 6 |
|  |  | A4ul, area 4(upper limb region) | −26, −25, 63 | 4 | 34, −19, 59 | 6 |
|  |  | A4t, area 4(trunk region) | −13, −20, 73 | 6 | 15, −22, 71 | 6 |
|  |  | A4tl, area 4(tongue and larynx region) | −52, 0, 8 | 6 | 54, 4, 9 | 6 |
|  |  | A6cvl, caudal ventrolateral area 6 | −49, 5, 30 | 6 | 51, 7, 30 | 6 |
|  | PCL, Paracentral lobule | A1/2/3ll, area 1/2/3 (lower limb region) | −8, −38, 58 | 5 | 10, −34, 54 | 5 |
|  |  | A4ll, area 4, (lower limb region) | −4, −23, 61 | 4 | 5, −21, 61 | 6 |
| Temporal lobe | STG, Superior temporal gyrus | A38m, medial area 38 | −32, 14, −34 | 38 | 31, 15, −34 | 38 |
|  |  | A41/42, area 41/42 | −54, −32, 12 | 22 | 54, −24, 11 | 41 |
|  |  | TE1.0 and TE1.2 | −50, −11, 1 | 41 | 51, −4, −1 | 41 |
|  |  | A22c, caudal area 22 | −62, −33, 7 | 21 | 66, −20, 6 | 41 |
|  |  | A38l, lateral area 38 | −45, 11, −20 | 38 | 47, 12, −20 | 38 |
|  |  | A22r, rostral area 22 | −55, −3, −10 | 22 | 56, −12, −5 | 22 |
|  | MTG, Middle temporal gyrus | A21c, caudal area 21 | −65, −30, −12 | 21 | 65, −29, −13 | 21 |
|  |  | A21r, rostral area 21 | −53, 2, −30 | 38 | 51, 6, −32 | 38 |
|  |  | A37dl, dorsolateral area 37 | −59, −58, 4 | 37 | 60, −53, 3 | 37 |
|  |  | aSTS, anterior superior temporal sulcus | −58, −20, −9 | 21 | 58, −16, −10 | 22 |
|  | ITG, Inferior temporal gyrus | A20iv, intermediate ventral area 20 | −45, −26, −27 | 20 | 46, −14, −33 | 20 |
|  |  | A37elv, extreme lateroventral area 37 | −51, −57, −15 | 37 | 53, −52, −18 | 37 |
|  |  | A20r, rostral area 20 | −43, −2, −41 | 20 | 40, 0, −43 | 20 |
|  |  | A20il, intermediate lateral area 20 | −56, −16, −28 | 20 | 55, −11, −32 | 20 |
|  |  | A37vl, ventrolateral area 37 | −55, −60, −6 | 37 | 54, −57, −8 | 37 |
|  |  | A20cl, caudolateral of area 20 | −59, −42, −16 | 37 | 61, −40, −17 | 37 |
|  |  | A20cv, caudoventral of area 20 | −55, −31, −27 | 20 | 54, −31, −26 | 20 |
|  | FuG, Fusiform gyrus | A20rv, rostroventral area 20 | −33, −16, −32 | 36 | 33, −15, −34 | 36 |
|  |  | A37mv, medioventral area 37 | −31, −64, −14 | 19 | 31, −62, −14 | 37 |
|  |  | A37lv, lateroventral area 37 | −42, −51, −17 | 37 | 43, −49, −19 | 37 |
|  | PhG, Parahippocampal gyrus | A35/36r, rostral area 35/36 | −27, −7, −34 | 36 | 28, −8, −33 | 36 |
|  |  | A35/36c, caudal area 35/36 | −25, −25, −26 | 36 | 26, −23, −27 | 36 |
|  |  | TL, area TL (lateral PPHC, posterior parahippocampal gyrus) | −28, −32, −18 | 37 | 30, −30, −18 | 36 |
|  |  | A28/34, area 28/34 (EC, entorhinal cortex) | −19, −12, −30 | 36 | 19, −10, −30 | 36 |
|  |  | TI, area TI(temporal agranular insular cortex) | −23, 2, −32 | 36 | 22, 1, −36 | 36 |
|  |  | TH, area TH (medial PPHC) | −17, −39, −10 | 19 | 19, −36, −11 | 36 |
|  | pSTS, Posterior superior temporal sulcus | rpSTS, rostroposterior superior temporal sulcus | −54, −40, 4 | 21 | 53, −37, 3 | 21 |
|  |  | cpSTS, caudoposterior superior temporal sulcus | −52, −50, 11 | 39 | 57, −40, 12 | 22 |
| Parietal lobe | SPL, Superior parietal lobule | A7r, rostral area 7 | −16, −60, 63 | 7 | 19, −57, 65 | 7 |
|  |  | A7c, caudal area 7 | −15, −71, 52 | 7 | 19, −69, 54 | 7 |
|  |  | A5l, lateral area 5 | −33, −47, 50 | 7 | 35, −42, 54 | 7 |
|  |  | A7pc, postcentral area 7 | −22, −47, 65 | 7 | 23, −43, 67 | 5 |
|  |  | A7ip, intraparietal area 7(hIP3) | −27, −59, 54 | 7 | 31, −54, 53 | 7 |
|  | IPL, Inferior parietal lobule | A39c, caudal area 39(PGp) | −34, −80, 29 | 19 | 45, −71, 20 | 19 |
|  |  | A39rd, rostrodorsal area 39(Hip3) | −38, −61, 46 | 39 | 39, −65, 44 | 39 |
|  |  | A40rd, rostrodorsal area 40(PFt) | −51, −33, 42 | 40 | 47, −35, 45 | 40 |
|  |  | A40c, caudal area 40(PFm) | −56, −49, 38 | 39 | 57, −44, 38 | 40 |
|  |  | A39rv, rostroventral area 39(PGa) | −47, −65, 26 | 39 | 53, −54, 25 | 39 |
|  |  | A40rv, rostroventral area 40(PFop) | −53, −31, 23 | 40 | 55, −26, 26 | 40 |
|  | Pcun, Precuneus | A7m, medial area 7(PEp) | −5, −63, 51 | 7 | 6, −65, 51 | 7 |
|  |  | A5m, medial area 5(PEm) | −8, −47, 57 | 7 | 7, −47, 58 | 7 |
|  |  | dmPOS, dorsomedial parietooccipital sulcus (PEr) | −12, −67, 25 | 18 | 16, −64, 25 | 31 |
|  |  | A31, area 31 (Lc1) | −6, −55, 34 | 31 | 6, −54, 35 | 31 |
|  | PoG, Postcentral gyrus | A1/2/3ulhf, area 1/2/3(upper limb, head and face region) | −50, −16, 43 | 1 | 50, −14, 44 | 4 |
|  |  | A1/2/3tonIa, area 1/2/3(tongue and larynx region) | −56, −14, 16 | 1 | 56, −10, 15 | 1 |
|  |  | A2, area 2 | −46, −30, 50 | 1 | 48, −24, 48 | 1 |
|  |  | A1/2/3tru, area 1/2/3(trunk region) | −21, −35, 68 | 1 | 20, −33, 69 | 1 |
| Insular lobe | INS, Insular gyrus | G, hypergranular insula | −36, −20, 10 | 13 | 37, −18, 8 | 13 |
|  |  | vIa, ventral agranular insula | −32, 14, −13 | 13 | 33, 14, −13 | 13 |
|  |  | dIa, dorsal agranular insula | −34, 18, 1 | 13 | 36, 18, 1 | 13 |
|  |  | vId/vIg, ventral dysgranular and granular insula | −38, −4, −9 | 13 | 39, −2, −9 | 13 |
|  |  | dIg, dorsal granular insula | −38, −8, 8 | 13 | 39, −7, 8 | 13 |
|  |  | dId, dorsal dysgranular insula | −38, 5, 5 | 13 | 38, 5, 5 | 13 |
| Limbic lobe | CG, Cingulate gyrus | A23d, dorsal area 23 | −4, −39, 31 | 23 | 4, −37, 32 | 23 |
|  |  | A24rv, rostroventral area 24 | −3, 8, 25 | 24 | 5, 22, 12 | 24 |
|  |  | A32p, pregenual area 32 | −6, 34, 21 | 32 | 5, 28, 27 | 32 |
|  |  | A23v, ventral area 23 | −8, −47, 10 | 30 | 9, −44, 11 | 30 |
|  |  | A24cd, caudodorsal area 24 | −5, 7, 37 | 24 | 4, 6, 38 | 32 |
|  |  | A23c, caudal area 24 | −7, −23, 41 | 31 | 6, −20, 40 | 24 |
|  |  | A32sg, subgenual area 32 | −4, 39, −2 | 32 | 5, 41, 6 | 32 |
| Occipital lobe | MVOcC, MedioVentral occipital cortex | cLinG, caudal lingual gyrus | −11, −82, −11 | 18 | 10, −85, −9 | 18 |
|  |  | rCunG, rostral cuneus gyrus | −5, −81, 10 | 17 | 7, −76, 11 | 17 |
|  |  | cCunG, caudal cuneus gyrus | −6, −94, 1 | 18 | 8, −90, 12 | 18 |
|  |  | rLinG, rostral lingual gyrus | −17, −60, −6 | 19 | 18, −60, −7 | 19 |
|  |  | vmPOS,ventromedial parietooccipital sulcus | −13, −68, 12 | 17 | 15, −63, 12 | 17 |
|  | LOcC, Lateral occipital cortex | mOccG, middle occipital gyrus | −31, −89, 11 | 18 | 34, −86, 11 | 18 |
|  |  | V5/MT+, area V5/MT+ | −46, −74, 3 | 19 | 48, −70, −1 | 37 |
|  |  | OPC, occipital polar cortex | −18, −99, 2 | 18 | 22, −97, 4 | 18 |
|  |  | iOccG, inferior occipital gyrus | −30, −88, −12 | 18 | 32, −85, −12 | 19 |
|  |  | msOccG, medial superior occipital gyrus | −11, −88, 31 | 18 | 16, −85, 34 | 19 |
|  |  | lsOccG, lateral superior occipital gyrus | −22, −77, 36 | 7 | 29, −75, 36 | 7 |
| Subcortical nuclei | Amyg, Amygdala | mAmyg, medial amygdala | -19, -2, -20 | NA | 19, -2, -19 | NA |
|  |  | lAmyg, lateral amygdala | -27, -4, -20 | NA | 28, -3, -20 | NA |
|  |  | lAmyg, lateral amygdala | -22, -14, -19 | NA | 22, -12, -20 | NA |
|  |  | cHipp, caudal hippocampus | -28, -30, -10 | NA | 29, -27, -10 | NA |
|  |  | vCa, ventral caudate | -12, 14, 0 | NA | 15, 14, -2 | NA |
|  |  | GP, globus pallidus | -22, -2, 4 | NA | 22, -2, 3 | NA |
|  |  | NAC, nucleus accumbens | -17, 3, -9 | NA | 15, 8, -9 | NA |
|  |  | vmPu, ventromedial putamen | -23, 7, -4 | NA | 22, 8, -1 | NA |
|  |  | dCa, dorsal caudate | -14, 2, 16 | NA | 14, 5, 14 | NA |
|  |  | dlPu, dorsolateral putamen | -28, -5, 2 | NA | 29, -3, 1 | NA |
|  |  | mPFtha, medial pre-frontal thalamus | -7, -12, 5 | NA | 7, -11, 6 | NA |
|  |  | mPMtha, pre-motor thalamus | -18, -13, 3 | NA | 12, -14, 1 | NA |
|  |  | Stha, sensory thalamus | -18, -23, 4 | NA | 18, -22, 3 | NA |
|  |  | rTtha, rostral temporal thalamus | -7, -14, 7 | NA | 3, -13, 5 | NA |
|  |  | PPtha, posterior parietal thalamus | -16, -24, 6 | NA | 15, -25, 6 | NA |
|  |  | Otha, occipital thalamus | -15, -28, 4 | NA | 13, -27, 8 | NA |
|  |  | cTtha, caudal temporal thalamus | -12, -22, 13 | NA | 10, -14, 14 | NA |
|  |  | lPFtha, lateral pre-frontal thalamus | -11, -14, 2 | NA | 13, -16, 7 | NA |
| MNI, Montreal Neurological Institute; NA, Not Applicable. | | | | | | |
