# Supplementary material for: Task-Dependent Effective Connectivity of the Reward Network During Food Cue-Reactivity: A Dynamic Causal Modeling Investigation
Source: Front Behav Neurosci. 2022 Jun 24;16:899605. doi: 10.3389/fnbeh.2022.899605 (PMC9263922; doi:10.3389/fnbeh.2022.899605)
Supplement: Supplementary file 5 [file Table_2.docx]

**SUPPLEMENTARY MATERIAL**

**Title:** Task-Dependent Effective Connectivity of the Reward Network During Food Cue-Reactivity: A Dynamic Causal Modelling Investigation

**Supplementary Table 2** **|** The results of the DCM PEB analyses for food cue-reactivity task.

| **Parameter*** | **Description** | **Units** | **Expectation** | **Variance** | **Posterior Probability** |
| --- | --- | --- | --- | --- | --- |
| $A_{I 11}$ | Self-connection on VTA | None | -0.6033 | 0.001012 | 1 |
| $A_{E 21}$ | VTA → lAmyg | Hz | 0.4839 | 0.002202 | 1 |
| $A_{E 31}$ | VTA → lOFC | Hz | 0.0422 | 0.004893 | 0.838968 |
| $A_{E 41}$ | VTA → rAmyg | Hz | 0.2689 | 0.003382 | 1 |
| $A_{E 51}$ | VTA → rOFC | Hz | 0.2282 | 0.004796 | 1 |
| $A_{E 12}$ | lAmyg → VTA | Hz | 0.3781 | 0.010792 | 1 |
| $A_{I 22}$ | Self-connection on lAmyg | None | -0.2547 | 0.001073 | 1 |
| $A_{E 32}$ | lAmyg → lOFC | Hz | 0.1002 | 0.002821 | 1 |
| $A_{E 42}$ | lAmyg → rAmyg | Hz | 0.1219 | 0.005071 | 1 |
| $A_{E 13}$ | lOFC → VTA | Hz | -0.3907 | 0.012591 | 1 |
| $A_{E 23}$ | lOFC → lAmyg | Hz | 0.4529 | 0.001857 | 1 |
| $A_{I 33}$ | Self-connection on lOFC | None | -0.5517 | 0.001076 | 1 |
| $A_{E 53}$ | lOFC → rOFC | Hz | 0.1349 | 0.001603 | 1 |
| $A_{E 14}$ | rAmyg → VTA | Hz | -0.2232 | 0.002845 | 1 |
| $A_{E 24}$ | rAmyg → lAmyg | Hz | -0.3433 | 0.019688 | 1 |
| $A_{I 44}$ | Self-connection on rAmyg | None | -0.16 | 0.001093 | 0.99969 |
| $A_{E 54}$ | rAmyg → rOFC | Hz | -0.0776 | 0.005381 | 0.999483 |
| $A_{E 15}$ | rOFC → VTA | Hz | 0.0049 | 0.012179 | 0 |
| $A_{E 35}$ | rOFC → lOFC | Hz | 0.1495 | 0.004155 | 1 |
| $A_{E 45}$ | rOFC → rAmyg | Hz | 0.3364 | 0.002197 | 1 |
| $A_{E 55}$ | Self-connection on rOFC | None | -0.4293 | 0.001004 | 1 |
| $B_{I 11}^{(1)}$ | Food images on VTA self-connection | None | 0.4936 | 0.131969 | 0.999846 |
| $B_{I 22}^{(1)}$ | Food images on lAmyg self-connection | None | -0.8268 | 0.090282 | 1 |
| $B_{I 33}^{(1)}$ | Food images on lOFC self-connection | None | 0.0041 | 0.104839 | 0 |
| $B_{I 44}^{(1)}$ | Food images on rAmyg self-connection | None | -0.364 | 0.055444 | 0.999842 |
| $B_{I 55}^{(1)}$ | Food images on rOFC self-connection | None | -0.5306 | 0.080635 | 1 |
| $C_{12}$ | Driving: food and neutral on VTA | None | -0.02 | 0.018372 | 0 |
| $C_{22}$ | Driving: food and neutral on lAmyg | None | 0.0655 | 0.020502 | 0 |
| $C_{32}$ | Driving: food and neutral on lOFC | None | 0.0025 | 0.016801 | 0 |
| $C_{42}$ | Driving: food and neutral on rAmyg | None | 0.019 | 0.01684 | 0 |
| $C_{52}$ | Driving: food and neutral on rOFC | None | 0.0324 | 0.014834 | 0 |

*Region names: 1=VTA, 2=left amygdala, 3=left OFC, 4=right amygdala, 5=right OFC. Condition names (superscript on matrix 𝐵𝐼): 1=Food cues. lAmyg, Left Amygdala; lOFC, Left Orbitofrontal Cortex; rAmyg, Right Amygdala; rOFC, Right Orbitofrontal Cortex; VTA, Ventral Tegmental Area.
